# In Vitro and In Vivo Antitumor Efficacy of *Hizikia fusiforme* Celluclast Extract against Bladder Cancer

**DOI:** 10.3390/nu12072159

**Published:** 2020-07-21

**Authors:** Jun-Hui Song, Se Yeon Won, Byungdoo Hwang, Soontag Jung, Changsun Choi, Sung-Soo Park, Yung Hyun Choi, Wun-Jae Kim, Sung-Kwon Moon

**Affiliations:** 1 Department of Food and Nutrition, Chung-Ang University, Anseong 17546, Korea; goodabc123@cau.ac.kr (J.-H.S.); wsy@vegemil.co.kr (S.Y.W.); byungdoo0409@naver.com (B.H.); amazing555@naver.com (S.J.); cchoi@cau.ac.kr (C.C.); 2 Department of Food Science and Nutrition, Jeju National University, Jeju 63243, Korea; foodpark@jejunu.ac.kr; 3 Department of Biochemistry, College of Oriental Medicine, Dongeui University, Busan 47340, Korea; choiyh@deu.ac.kr; 4 Department of Urology, Chungbuk National University, Cheongju, Chungbuk 361-763, Korea; wjkim@chungbuk.ac.kr

**Keywords:** *Hizikia fusiforme*, bladder cancer, EJ, cell cycle, JNK, migration, xenograft mice

## Abstract

Various physiological benefits have been linked to *Hizikia fusiforme* (HF), an edible brown seaweed. Here, fucose-containing sulfated polysaccharides were extracted from celluclast-processed HF (SPHF) and their antitumor efficacy against bladder cancer was evaluated in vitro and in vivo. SPHF possesses high sulfated polysaccharide and fucose contents and free radical scavenging activities compared to those of celluclast-processed HF extracts (CHF). SPHF inhibited bladder cancer EJ cell proliferation via G1-phase cell cycle arrest. This was due to the induction of p21WAF1 expression associated with the downregulation of CDKs and cyclins. Moreover, JNK phosphorylation was identified as an SPHF-mediated signaling molecule. SPHF treatment also hindered the migration and invasion of EJ cells by inhibiting MMP-9 expression, which was attributed to the repression of transcriptional binding to NF-κB, AP-1, and Sp-1 in the MMP-9 promoter region. In an animal study, SPHF treatment suppressed EJ tumor growth in xenograft mice similarly to cisplatin. Furthermore, no toxicity signs were found after weight loss assessment, biochemical tests, and organ tissue immunostaining during oral administration of 20–200 mg/kg SPHF for 20 days. Therefore, our study demonstrates the antitumor efficacy of SPHF in vitro and in vivo, thus highlighting its potential for bladder cancer treatment development.

## 1. Introduction

Urinary bladder cancer is among the most fatal cancer types in males and elderly people worldwide [1]. The majority of bladder cancer cases are transitional cell carcinomas, which are classified as superficial and muscle-invasive lesions [2,3]. Superficial lesions occurring in approximately 70% of bladder tumor patients are typically controlled with chemotherapy, adjuvant therapy, and transurethral resection [4]. However, muscle-invasive lesions are linked to low survival rates and poor prognosis as current treatments cannot effectively prevent metastatic transition, which ultimately leads to death [5,6]. Therefore, novel therapies for bladder cancer treatment are urgently needed.

During neoplastic tumor progression, bladder cancer cells proliferate and metastasize by triggering a multistep process via signaling cascades, cell cycle regulation, and migratory and invasive modulation [7]. Transmission of extracellular signaling including mitogen-activated protein kinases (MAPKs) and PI3K/AKT leads to an uncontrolled cell cycle progression through activation of cell-cycle regulators such as cyclin-dependent kinases (CDKs), cyclins, and CDK inhibitors (CKIs) in the G1, S, and G2/M cell-cycle phases [8,9,10,11,12,13]. The positive regulatory proteins CDK2, CDK4, and cyclins (cyclin D1 and cyclin E) and the negative regulator p21WAF1 have been found to modulate the G1- to S-phase cell cycle progression and have emerged as promising potential targets for chemotherapeutic antitumor agents [12,13]. Additionally, the migration and invasion of cancer cells induced by matrix metalloproteinases (MMPs) is also a promising target for the treatment of bladder tumors [8,14,15,16]. MMP-9 activity is responsible for migratory and invasive potential and is deeply associated with bladder cancer progression [14,15,16]. Specifically, MMP-9 regulation is positively modulated via the binding activity of transcription factors NF-κB, Sp-1, and AP-1 during tumor-associated migration and invasion [17,18,19].

*Hizikia fusiforme* (HF) is an edible brown seaweed that inhabits the northwest Pacific, including China, Japan, and Korea [20]. During food processing, HF byproducts extracted from water are typically discarded. However, these byproducts contain several types of sulfated polysaccharides [21], which have been found to possess various beneficial properties [20,21]. Previous studies have revealed that HF polysaccharides possess antioxidant, anti-inflammatory, anti-diabetic, osteoprotective, and immunostimulatory effects [21,22,23,24,25]. Notably, a recent report demonstrated that sulfated polysaccharides extracted from acid-processed HF possessed antitumor effects in vitro [26,27]. However, the molecular mechanisms by which these compounds exert antitumor effects have not been sufficiently explored in vitro or in animal models. Therefore, this study examined the in vitro and in vivo antitumor activity of fucose-containing sulfated polysaccharides extracted from enzyme-processed HF (SPHF) in human bladder cancer cells.

## 2. Materials and Methods

### 2.1. Materials

Anti-extracellular signal-regulated kinase (ERK), anti-phospho-ERK, anti-p38 mitogen-activated protein kinase (MAPK), anti-phospho-p38 MAPK, anti-Janus kinase (JNK), anti-phospho-JNK, anti-protein kinase B (AKT), and anti-phospho-AKT antibodies were purchased from Cell Signaling Technology Inc. (Danvers, MA, USA). Polyclonal anti-cyclin D1, anti-cyclin E, anti-cyclin-dependent kinase (CDK) 2, anti-CDK4, anti-p53, anti-p21WAF1, anti-p27KIP1, and anti-GAPDH antibodies were obtained from Santa Cruz Biotechnology Inc. (Santa Cruz, CA, USA). Moreover, a Ki-67-specific antibody was obtained from Invitrogen (Waltham, MA, USA). Moreover, a nuclear extract kit and electrophoretic mobility shift assay (EMSA) gel shift kit were purchased from Panomics (Fremont, CA, USA). SP600125 were purchased from Calbiochem (San Diego, CA, USA).

### 2.2. Preparation of Sulfated Polysaccharides from Hizikia fusiforme (SPHF)

*H. fusiforme* (HF) was collected in the coast of Jeju Island, South Korea. The sample was washed with water to remove surrounding salt and then boiled in water at 100 °C for 5 min to eliminate arsenic, then freeze-dried and ground to a powder. 10 g of lyophilized seaweed powder were then mixed with 1 L of distilled water, after which the pH was adjusted with 1 M HCl. Afterward, 500 μL of Celluclast was added to the mixture to obtain a final 5% enzyme to seaweed ratio. Finally, enzyme-assisted extraction was performed under constant shaking at 50 °C for 24 h. After hydrolysis, the enzyme was inactivated by heating at 100 °C for 10 min. After centrifugation and filtering, lysates were adjusted to a pH of 7 by adding 1 M NaOH. A final celluclast-assisted HF extract was then obtained and will hereinafter be referred to as CHF. An aqueous CaCl_2_ solution was added to precipitate the alginates in the HF in the form of calcium alginate, given that these alginates are considered contaminants during the FCSP (fucose-containing sulfated polysaccharide) isolation process. The remaining debris was clarified through centrifugation. The supernatant pH was adjusted to 7.0 making it a neutral solution and then lyophilized to concentrate the solution to one-third of its original volume. The polysaccharides were then precipitated by the addition of 4 initial volumes of 95% ethanol, after which the mixture was maintained at 4 °C for 8 h. The precipitated polysaccharides were recovered by centrifugation at 16,000× g for 10 min and were homogenized by dissolving in distilled water. The preparation was then lyophilized to obtain a powder that will henceforth be referred to as “sulfated polysaccharides extracted from celluclast-assisted HF” (SPHF). SPHF was dissolved in water for the experiments.

### 2.3. CHF and SPHF Chemical Analysis

The total phenolic contents of the CHF and SPHF extracts were determined using the modified Folin-Denis method [28] and the results were presented as gallic acid concentration equivalents. To estimate the total sulfate content, CHF and SPHF were hydrolyzed by adding 4 M trifluoroacetic acid at 100 °C for 5 h. The sulfate content was then measured as described by Kang et al. [29,30]. The monosaccharide composition of the polysaccharides was analyzed following acid hydrolysis using 6 N of HCl for 4 h. The analysis was carried out using a CarboPac PA1 cartridge column (4.5 × 50 mm) integrated to an ED50 Dionex Electrochemical Detector as described by Lee et al. [29,30] using fucose as the standard.

### 2.4. HF and HFPS Antioxidant Assay

The radical scavenging activity of CHF and SPHF were evaluated using DPPH and ABTS scavenging assays. For the DPPH assay, 0.1 mL of sample extract with varying concentrations was added to the same volume of a DPPH methanolic solution. The mixtures were then shaken well with a vortex mixer and kept at room temperature for 20 min. Changes in the absorbance of the extract samples were measured at 520 nm using a UV-visible spectrophotometer. For the ABTS assay, ABTS was dissolved in water to a 7 μM concentration and radical cations (ABTS+) were produced by allowing the ABTS solution to react with 2.45 μM potassium persulfate at room temperature in the dark (18 h) before use. To perform the assays, the ABTS+ solution was diluted with water to an absorbance value of 0.700 ± 0.02 at 734 nm. Absorbance was then recorded 6 min after adding 3.0 mL of diluted ABTS+ solution to 100 μL of extracts solutions.

### 2.5. Cell Culture and Treatment

The human bladder cancer EJ (MGH-U1) cell line examined herein was provided by Dr. Wun-Jae Kim (Department of Urology, Chungbuk National University; Chungbuk, South Korea). EJ cells were maintained in Dulbecco’s modified Eagle’s medium (DMEM) supplemented with 10% fetal bovine serum (FBS) and 1% penicillin/streptomycin (Gibco, New York, NY, USA) at 37 °C in a 5% CO_2_ humidified incubator. In addition, the human normal bladder fibroblast cell (BdFC) line was used to examine the effect of SPHF. BdFC cells were obtained from American Type Culture Collection (ATCC, Baltimore, MD, USA) and maintained in fibroblast basal medium with supplements provided by the manufacturer (ATCC, Baltimore, MD, USA). Cells were grown in the presence of 5% CO_2_ in air at 37 °C. The cells were then treated with various concentrations of SPHF for 24 h once confluency reached approximately 70%.

### 2.6. Viability Assays

Cells (3 × 10^3^/well) were seeded in 96-well plates and incubated overnight at 37 °C. The cells were then treated with various concentrations of SPHF for 24 h. Afterward, a 3-(4,5-Dimethylthiazol-2-yl)-2,5-diphenyltetrazolium bromide (MTT) assay, CCK-8 assay, and cell counting assay were performed as reported in a previous study [19]. One representative graph out of three independent experiments was presented in the results.

### 2.7. Cell Cycle Analysis

Cell cycle phases were identified with a MUSE^®^ Cell Analyzer and analysis software (Merck Millipore, Burlington, MA, USA) using a Muse^®^ Cell Cycle Assay Kit (Merck Millipore, Burlington, MA, USA) according to the manufacturer’s instructions.

### 2.8. Immunoblots and Immunoprecipitation

EJ cells (8 × 10^5^ cells) were seeded in 100 mm cell culture dishes and treated with various concentrations of SPHF for 24 h. Immunoblots and immunoprecipitation were performed as described previously [19].

### 2.9. Wound-Healing Migration Assays

EJ cells were plated in 6-well plates and grown to 90% confluence in 2 mL of growth medium and a line-shaped incision to the confluent monolayer of growth-arrested cells was generated using a 2 mm pipette tip. The cells were treated with various concentrations of SPHF for 24 h and allowed to migrate into the scraped area. Images were captured using an inverted microscope (40 x magnification; Optika, Ponteranica, Italy).

### 2.10. Invasion Assays

EJ cells (2.5 × 10^4^ cells) were resuspended with various concentrations of SPHF in 100 μL of medium and placed in the upper part of a trans-well plate. The cells had to pass through a polycarbonate membrane with 8 μm-sized pores and a thin layer of an ECM matrix-like material. The ability of the cells to invade the ECM matrix-like material was determined using a commercial cell invasion assay kit (Chemicon International Inc., Billerica, MA, USA).

### 2.11. Zymography

The bladder cancer EJ cells cultured in 6-well plates were treated with various concentrations of SPHF for 24 h. The conditioned medium was electrophoresed on a polyacrylamide gel containing 1 mg/mL gelatin (Sigma-Aldrich, St. Louis, MO, USA). The gel was then washed at room temperature for 2 h with 2.5% Triton X-100 and maintained at 37 °C overnight in a pH 7.5 buffer containing 10 mM CaCl_2_, 150 mM NaCl, and 50 mM Tris-HCl (Sigma-Aldrich, St. Louis, MO, USA). The gel was stained with 0.2% Coomassie blue (Bio–Rad Laboratories, Hercules, CA, USA) and images were captured using a lightbox (Matin International, Seoul, Korea). Proteolysis was detected as a white area in a dark blue field with the ImagePro Plus 6.0 software (Media Cybernetics, Bethesda, MD, USA).

### 2.12. Nuclear Extracts and Electrophoretic Mobility Shift Assay

Nuclear extracts were prepared and subjected to an electrophoretic mobility shift assay (EMSA) for measurement of AP-1, Sp-1, and NFκB activities, as reported previously [19].

### 2.13. Animals

Six-week-old male BALB/c nude mice were purchased from the Dae-Han Experimental Animal Center (Dea-Han Biolink Co., Chungbuk, Korea). The rats were housed under the following controlled environmental conditions: constant temperature of 25 ± 2 °C, 60 ± 10% humidity, a 12 h light-dark cycle, and an ad libitum standard pellet diet (Deanhan Biolink Co., Chungbuk, Korea) and drinking water. All experiments were reviewed and approved by the Chungbuk National University Institutional Animal Care and Use Committee (IACUC). (Approval Number: EBOA-2015-08).

### 2.14. Mouse Xenograft Generation

To determine the inhibitory efficacy of SPHF in vivo, xenografts were established in BALB/c nude mice. EJ cells (1 × 10^7^ cells/mouse) were injected subcutaneously into the right flank of each mouse. The mice were then randomly divided into four groups (*n* = 5 each). Mice in the negative control group (Con) and BPH group were administered distilled water as a vehicle, whereas mice in the positive control group were administered 5 mg/kg Cisplatin (Merck Sharp & Dohme, Rahway, NJ, USA). Finally, mice in the SPHF group were administered 20 and 200 mg/kg. Bodyweight and tumor volume were measured once a day during the experiment. After the experiment, the mice were euthanized with CO_2_ gas, after which tumor masses were harvested. Tumor volume and weight were measured again, and tumor tissues were divided in half; one-half was fixed in 10% formalin and embedded in paraffin for histomorphological assays, whereas the other half was stored at −80 °C.

### 2.15. Immunohistochemistry

Tumor tissues as well as liver, heart, kidney, and lung samples were obtained from xenograft mice and then fixed in 10% formalin (Sigma-Aldrich, St. Louis, MO, USA), after which the samples were dehydrated in ethanol and embedded in paraffin blocks. Then, 5-μm paraffin sections were stained with H&E and Ki-67. Tissue sections were visualized and photographed with a fluorescence microscope.

### 2.16. Plasma Preparation and Biochemical Analysis

Collected blood samples remained at room temperature for 2 h and serums were separated by centrifuging at 3000× *g* for 40 min at 4 °C. Plasma biochemical parameters such as aspartate aminotransferase (AST), alanine aminotransferase (ALT), alkaline phosphatase (ALP), urea, and creatinine were analyzed with commercial kits (Abcam, Burlingame, CA, USA).

### 2.17. Statistical Analysis

All data were presented as the mean ± standard deviation (SD). The data were analyzed via factorial analysis of variance (ANOVA) and Fisher’s least significant difference test. A *p* < 0.05 value was deemed to represent a statistically significant difference. Statistical analysis was performed using the SPSS Statistics 18.0.1 software (IBM, Chicago, IL, USA).

## 3. Results

### 3.1. Total Phenolic Content, Sulfated Polysaccharide Content, Fucose Content, and Free Radical Scavenging Activities of CHF and SPHF

CHF and SPHF yields were determined to be 45.12% and 31.2%, and the total polyphenol contents of CHF and SPHF were 1.88 and 1.79 µg gallic acid/mL, respectively (Table 1). Additionally, the CHF and SPHF sulfated polysaccharide contents were 51.32% and 65.23%, and the proportions of fucose content in CHF and SPHF monosaccharides were 27.31% and 59.63% (Table 1). Table 2 summarizes the antioxidant evaluation results, as measured by the capacity to scavenge 2,2-diphenyl-1-picrylhydrazyl (DPPH) and to inhibit lipid peroxidation events (TBARS assay). Both HF and SPHF exhibited strong antioxidant activity (Table 2). Particularly, SPHF appeared to have a superior radical scavenging activity compared to HF (Table 2). We therefore selected SPHF for downstream antitumor effect experiments. 

### 3.2. SPHF Inhibits Bladder Cancer EJ Cell Proliferation via Induction of G1-Phase Cell Cycle Arrest

**Figure 1:**
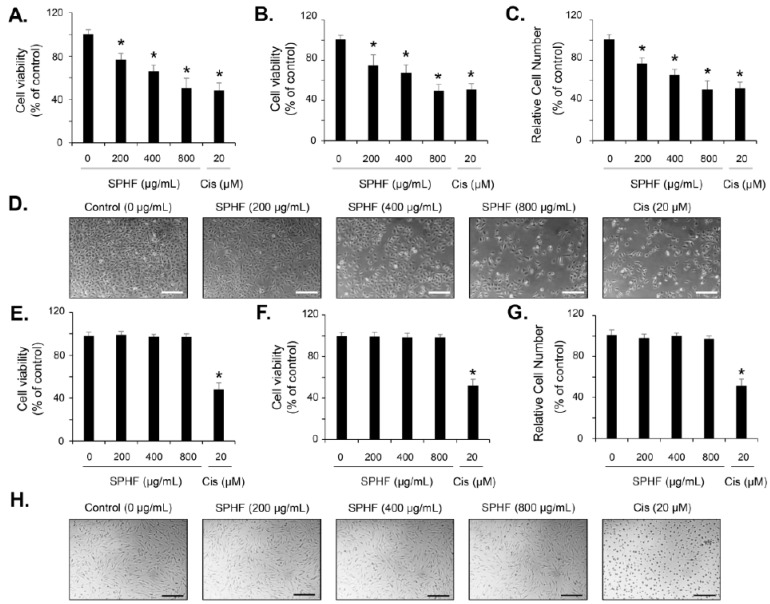
SPHF inhibited the proliferation of bladder cancer EJ cells. Both bladder cancer EJ cells and normal BdFC cells were treated with various concentrations of SPHF for 24 h. Both the MTT assay (**A**,**E**) and CCK-8 assay (**B**,**F**) were used to detect cell viability. (**C**,**G**) Cell numbers were counted via Trypan Blue staining. (**D**,**H**) Morphologic changes were photographed at the indicated SPHF treatment concentrations in EJ and BdFC cells (scale bars = 100 µm). Cisplatin was used as a positive control (**A**–**H**). Values are presented as the mean ± SD of three independent experiments; * *p* < 0.05 relative to the non-treated group.

**Figure 2:**
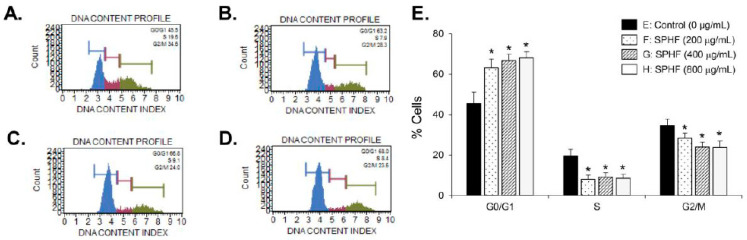
SPHF induced G1-phase cell cycle arrest in EJ cells. (**A**–**D**) FACS histograms of EJ cells followed by different SPHF treatment concentrations. (**E**) Cell cycle distribution in SPHF-treated cell populations. Values are presented as the mean ± SD of three independent experiments; * *p* < 0.05 relative to the non-treated group.

To investigate the antitumor properties of SPHF in bladder cancer cells, we assessed the inhibitory effect of SPHF on human bladder carcinoma EJ and normal BdFC cell proliferation. SPHF treatment reduced EJ cell viability in a dose-dependent manner according to both the MTT and CCK-8 assays (Figure 1A,B). The number of viable cells identified via Trypan Blue staining was similar to those determined by the MTT and CCK-8 assays after SPHF treatment (Figure 1C). In addition, an IC_50_ value of 800 μg/mL was determined by the proliferation assay (Figure 1A–C). A decreased cell number was clearly observed in SPHF-treated cells, as demonstrated by microscope examination (Figure 1D). In addition, the cytotoxicity of SPHF to normal BdFC cells was not observed, as evaluated by MTT, CCK-8, and viable cell counting assays, compared with EJ cells (Figure 1E–G). Similar result was found in cell morphology using microscopic observation (Figure 1H). Cisplatin, a known chemotherapeutic drug, was also used as a positive control to assess the antiproliferative effect of SPHF. The growth inhibitory effect of SPHF (800 μg/mL) in EJ cells was almost equivalent to that of 20 μM cisplatin (Figure 1A–D). Moreover, antiproliferative effect of cisplatin (20 μM) was observed in BdFC cells (Figure 1E–H). After certifying the antiproliferative effect of SPHF in EJ cells, the cell cycle distribution assay was performed. FACS analysis demonstrated a significant accumulation of SPHF-treated cells in the G1-phase (Figure 2A–E). In line with the FACS result, cell populations in the S and G2/M phase decreased after SPHF treatment (Figure 2A–E).

### 3.3. SPHF-Induced Inhibition of Cell Proliferation was Involved in the p21WAF1-Mediated G1-Phase Cell Cycle Arrest via Decreased Expression of CDKs and Cyclins

**Figure 3:**
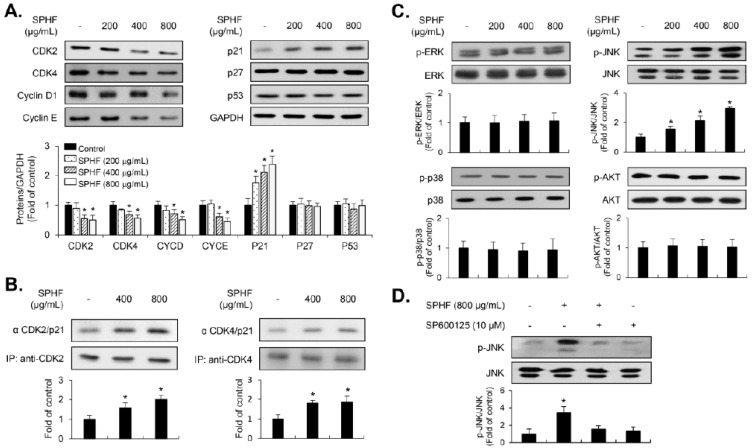
Involvement of cell-cycle regulators and signaling molecules during SPHF-induced suppression of EJ cell proliferation. EJ cells were treated with the indicated concentrations of SPHF for 24 h. (**A**) Immunoblot analysis of cell cycle-associated proteins in SPHF-treated EJ cells. GAPDH was utilized as an internal control. (**B**) Cell lysates were immunoprecipitated with anti-CDK2 and anti-CDK4 antibodies, after which immunoblot analysis was performed using an anti-p21WAF1 antibody. (**C**) Phosphorylation levels of MAPKs (ERK1/2, JNK, and p38MAPK) and AKT were measured via immunoblot analysis. (**D**) JNK phosphorylation specificity was evaluated via immunoblot analysis using its specific kinase inhibitor SP600125. The bar graphs present fold changes in expression levels compared to the controls. Values are presented as the mean ± SD of three independent experiments; * *p* < 0.05 relative to the non-treated group.

Expression levels of G1-phase cell cycle-related proteins in SPHF-treated EJ cells were measured to evaluate the mechanisms by which SPHF affects cell proliferation. CDK2 and CDK4, as well as their compatible partners in the G1-phase cyclin E and cyclin D1, were downregulated after SPHF treatment (Figure 3A). In contrast, SPHF treatment upregulated p21WAF1 expression (Figure 3A). However, SPHF treatment did not change the expression level of either p27KIP1 or p53 (Figure 3A). Given that p21WAF1 is a pivotal CDK regulator during G1 to S transition, immunoprecipitation assays were performed using anti-CDK2 and anti-CDK4 antibodies, followed by immunoblotting with anti-p21WAF1 antibodies. Immunoprecipitation data revealed that SPHF significantly enhanced p21WAF1 binding to both CDK2 and CDK4 in EJ cells (Figure 3B).

### 3.4. JNK Signaling is Associated with SPHF-Induced Inhibition of EJ Cell Proliferation

MAPKs (ERK1/2, p38MAPK, and JNK) and AKT signaling are reportedly involved in mediating bladder cancer progression and development [8,9,10,11]. Therefore, we also investigated the phosphorylation level of MAPKs and AKT signaling molecules in SPHF-treated EJ cells. SPHF treatment increased JNK phosphorylation in EJ cells (Figure 3C). However, the phosphorylation levels of both ERK1/2 and p38MAPK remained largely unaffected by SPHF treatment (Figure 3C). Additionally, the level of AKT phosphorylation also remained largely constant (Figure 3C). A JNK inhibitor (SP600125) was then used to further investigate the direct effect of SPHF on JNK signaling. This experiment determined that SP600125 treatment completely inhibited SPHF-induced JNK phosphorylation (Figure 3D).

### 3.5. SPHF Inhibits Migration and Invasion of EJ Cells via Decreased MMP-9 Expression Mediated by Suppression of Transcription Factor AP-1, Sp-1, and NF-κB Binding Activity

**Figure 4:**
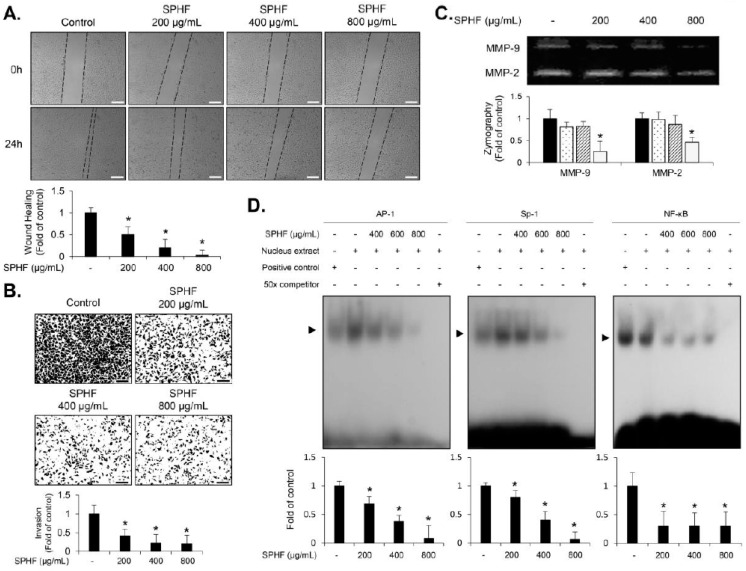
SPHF inhibited EJ cell migration and invasion via repression of MMP-9 activity by attenuating the binding activity of transcription factors. The cells were treated with different concentrations of SPHF for 24 h. (**A**) The inhibitory effects of SPHF on cellular migration were determined using a wound-healing migration assay (scale bars = 200 µm). (**B**) Invasive potential was evaluated with Matrigel-coated transwell plates in SPHF-treated cells (scale bars = 200 µm). (**C**) Changes in MMP-2 and MMP-9 activity were investigated via SPHF-treated cell zymography. (**D**) The EMSA assay was used to determine the transcriptional binding activity of NF-κB, AP-1, and Sp-1. The values in the bar graphs represent the mean ± SD of three independent experiments and all fold changes are relative to the control; * *p* < 0.05 relative to the non-treated group.

Matrix metalloproteins (MMPs) have been identified as crucial molecules for extracellular matrix degradation, which leads to cancer cell migration and invasion [8,14,15,16]. To examine the effect of SPHF migratory and invasive potential in EJ cells, wound-healing migration assays and Boyden chamber assays were performed. SPHF treatment significantly inhibited the migratory closure of EJ cells in a concentration-dependent manner (Figure 4A). Moreover, the invasive ability of EJ cells was also suppressed by SPHF treatment (Figure 4B). Additionally, SPHF reduced both MMP-9 and MMP-2 enzymatic activity in EJ cells (Figure 4C). We next investigated MMP-9 regulation in SPHF-treated EJ cells using the EMSA assay due to the known role of MMP-9 expression in bladder cancer migration and invasion [14,15,16]. Based on the EMSA assay results, SPHF blocked the binding activities of transcription factors NF-κB, Sp-1, and AP-1, which are cis-elements that comprise the MMP-9 promoter region (Figure 4D).

### 3.6. SPHF Suppressed EJ Bladder Cancer Cell Xenografted Tumors without Apparent Toxicity

**Figure 5:**
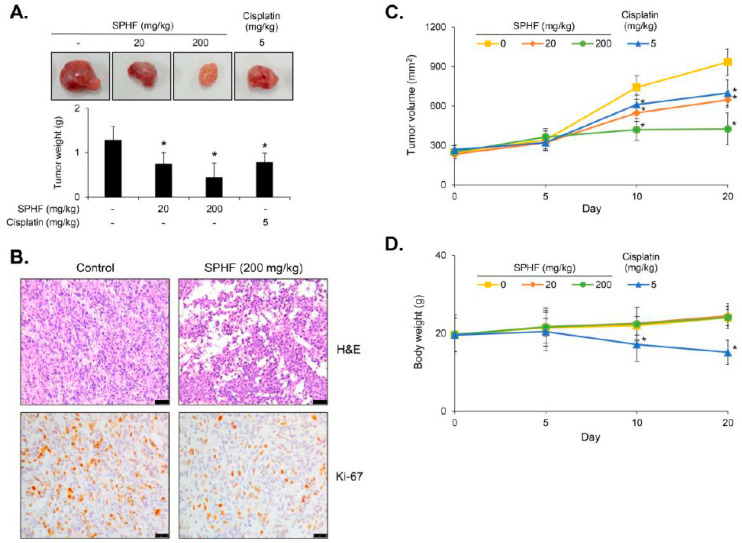
SPHF inhibited tumor growth in xenograft mice implanted with EJ cells. SPHF was orally administered at the indicated concentrations. Cisplatin (5 mg/kg) was also employed as a positive control to compare antitumor activity. (**A**) Weights and appearance of tumors isolated from EJ cell xenograft mice. (**B**) Tumor growth was evaluated by H&E and Ki-67 staining (scale bars = 50 µm). (**C**) Tumor volumes obtained from xenograft mice were assessed daily. (**D**) Body weights were evaluated during SPHF administration (0, 20, and 200 mg/kg) and compared to cisplatin (5 mg/kg) treatment. Values are presented as the mean ± SD of three independent experiments; *n* = 5, * *p* < 0.05 relative to the non-treated group.

**Figure 6:**
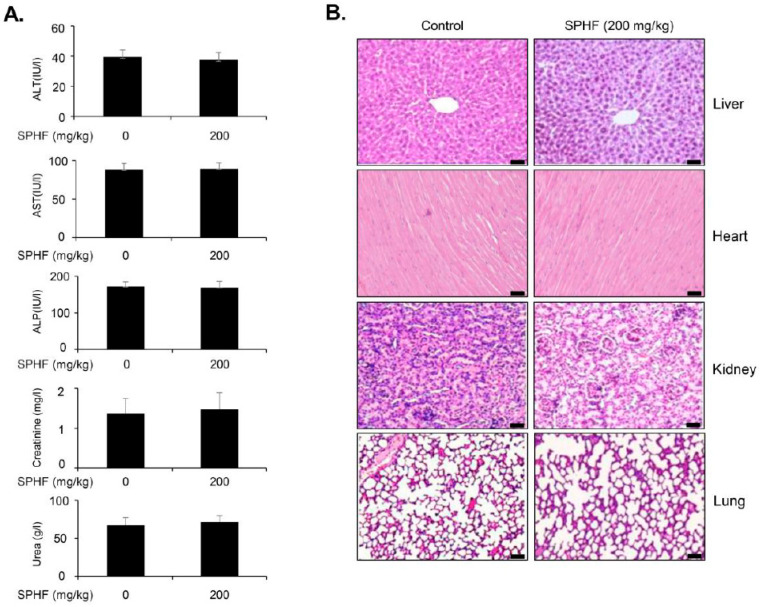
Effect of SPHF on biochemical parameters and H&E staining in EJ cell xenograft mice. (**A**) Biochemical factor (AST, ALT, ALP, urea, and creatinine) levels were estimated in SPHF-treated and non-treated xenograft mice. (**B**) Photomicrograph analysis of H&E staining in major organs (liver, kidney, lung, and heart, scale bars = 50 µm).

To analyze the efficacy of SPHF in vivo, different concentrations of SPHF (0, 20, and 200 mg/kg) were administered to Balb/C nude mice bearing EJ tumor xenografts via oral gavage. Cisplatin was also administered as a positive control to evaluate the antitumor potency and safety of SPHF. Daily oral gavage of 20–200 mg/kg SPHF administered for 4 weeks significantly repressed tumor weight in EJ bladder cancer xenografts (Figure 5A). H&E and Ki-67 tumor tissue staining revealed a prominent decline in the number of cancer cells in tumors treated with SPHF (Figure 5B). Additionally, upon 20 mg/kg SPHF treatment, tumor volume increased gradually after five days of administration (Figure 5C). However, the 200 mg/kg SPHF dose did not result in any noticeable changes for up to 10 days (Figure 5C). Importantly, our results demonstrated that the effect of 20 mg/kg SPHF on tumor reduction (weight and volume) was similar to that of cisplatin (Figure 5A,C). Mice body weight was then measured to identify potential SPHF side effects. Weight loss was not observed in SPHF-treated mice, whereas cisplatin treatment resulted in an approximately 20% weight loss in mice (Figure 5D). Finally, toxicity tests were also performed using plasma biochemical analysis and histologic evaluation of organ tissues obtained from both SPHF-treated and non-treated mice. The levels of ALT, AST, ALP, creatinine, and urea were unaffected by oral SPHF administration (Figure 6A). Moreover, organ tissues from SPHF-treated mice, including liver, heart, kidney, and lung, did not exhibit significant pathological signs compared with non-treated mice (Figure 6B).

## 4. Discussion

*Hizikia fusiforme* (HF) is a widely-known edible brown seaweed that contains polysaccharides, vitamins, and phenolic compounds, all of which are associated with numerous physiological benefits [20,21,22,23,24,25,26,27]. Particularly, polysaccharide-enriched HF has recently attracted attention due to its diverse therapeutic properties [20,21,22,23,24,25,26,27]. Previous studies have demonstrated the antitumor effect of polysaccharide-enriched HF in vitro [26,27]. However, the antitumor efficacy of fucose-containing sulfated polysaccharides extracted from enzyme-processed HF (SPHF) has not been characterized. Therefore, this study sought to investigate the exact antitumor mechanisms of SPHF in vitro and in vivo against bladder cancer. Moreover, we also verified whether SPHF exerted toxic effects by measuring relevant biological parameters and performing tissue-organ immunohistology.

HF was boiled, hydrolyzed with Celluclast (CHF), and precipitated with CaCl_2_, followed by further precipitation with ethanol (SPHF), after which CHF and SPHF composition was analyzed. SPHF was found to contain a higher sulfated polysaccharide fraction compared to CHF. Furthermore, the phenolic content of SPHF was slightly lower than that of CHF. These results indicated that sulfated polysaccharide became concentrated, whereas phenolic content was eliminated during CaCl_2_ and ethanol precipitation. Monosaccharide composition assays revealed a higher amount of fucose in SPHF than in CHF. Previous studies have demonstrated that high levels of sulfated polysaccharide and fucose are linked to stronger antioxidant activities [20,23,31]. Additionally, the sulfating process has been found to enhance the physiological bioactive effects of polysaccharides [20,31]. In agreement with previous studies, our results demonstrated that SPHF possessed a stronger antioxidant activity than its non-sulfated counterparts due to their high sulfated carbohydrate and fucose contents. Therefore, subsequent antitumor efficacy studies focused exclusively on SPHF.

Uncontrolled abnormal proliferation of cancer cells plays an important role in the progression and development of bladder tumors. Many studies have demonstrated that inhibition of tumor cell proliferation was deeply associated with cell cycle regulation [12,13]. Many kinds of antitumor chemotherapy reagents have been developed, which primarily act as inhibitors of cell cycle progression [12,32,33]. Therefore, the control of cell cycle regulators that drive critical processes at specific cell cycle transition stages may be an effective basis for the development of anticancer agents [12,13,32,33]. In the present study, SPHF treatment inhibited the proliferation of bladder cancer EJ cells in a dose-dependent manner. The potential growth inhibitory effect of SPHF (800 μg/mL) was similar to that of 20 μM cisplatin. Cytotoxicity of SPHF toward normal BDFCs was negligible up to 800 μg/mL. SPHF-induced inhibition of EJ cell proliferation was attributed to the accumulation of G1-phase-arrested cells via downregulation of cyclin D1/CDK4 and cyclin E/CDK2 mediated by p21WAF1 upregulation. This novel and valuable observation indicates that cell cycle transition regulators may be used as molecular markers for SPHF-based clinical applications. It has been suggested that the phosphorylation of MAPKs (ERK1/2, JNK, and p38MAPK) and AKT is involved in various cellular events that regulate cell growth, survival, cell growth retardation, and cell cycle [9,10,11,34,35]. Although the importance and role of signaling pathways in HF extract-induced inhibition of cell growth have been increasingly acknowledged, this topic remains largely understudied. Only one study has reported ERK1/2 phosphorylation increases associated with HF-stimulated osteogenic effects upon administration of polysaccharide-enriched byproducts [21]. Importantly, our study for the first time demonstrated the SPHF-induced phosphorylation of JNK in EJ bladder cancer cells. Differences in HF- and SPHF-mediated signaling pathways are likely dependent on sulfated polysaccharide and fucose levels. The present study suggests that JNK signaling is a crucial regulator of SPHF-stimulated inhibition of EJ cell proliferation via cell-cycle progression blockade.

In this study, the migratory and invasive capacity of EJ cells was effectively repressed by SPHF treatment. A previous study reported that an HF ethanol extract suppressed invasion of HepG3 human hepatocarcinoma cells and found that major components of TJ (claudins-1, -3, and -4) and the insulin-like growth factor-1 receptor were simultaneously inhibited [36]. The metastasis of migratory and invasive cells to the adjacent muscle layer lining the bladder is an essential factor for bladder cancer progression [7,8,14]. Many studies support the notion that MMP-9 is closely linked to bladder cancer migratory and invasive capacity [15,16,19]. MMP-9 degrades the extracellular matrix (ECM) via proteolysis, which in turn promotes the migration and invasion of tumor cells [15,16,17,18,19]. Additionally, MMP-9 expression in migratory and invasive tumor cells is thought to be regulated by transcription factors NF-κB, AP-1, and Sp-1, which are located in its promoter region [17,18,19]. A previous study reported that HF extract effectively protected collagen synthesis and reduced the expression of MMPs by regulating both NF-κB and AP-1 protein levels in UVB-irradiated HDF cells [20]. In the present study, SPHF significantly inhibited MMP-9 gelatinase activity in EJ bladder cancer cells. We also found that SPHF treatment led to a reduction in NF-κB, AP-1, and Sp-1 transcriptional activity. The ability of SPHF to impede transcription-factor-mediated MMP-9 expression suggests that it likely disrupts the migration and invasion of bladder tumors. Future studies should therefore examine the role and function of MMP-2 in SPHF-induced inhibition of bladder cancer metastasis.

The antitumor efficacy of SPHF was assessed in vivo by subcutaneously injecting bladder cancer EJ cells into a mouse xenograft model. Here, both tumor weight and volume in xenografted mice were suppressed by SPHF treatment in a dose-dependent manner. Decreased proliferation of tumor cells was validated by immunohistochemical (IHC) detection of H&E and Ki-67. Our study also revealed that the potential antitumor effect of 20 mg/kg SPHF was equivalent to that of 5 mg/kg cisplatin. However, cisplatin treatment (5 mg/kg) was associated with weight loss, which was interpreted as a potential adverse side effect. In contrast, SPHF doses as high as 200 mg/kg had no apparent effects on body weight. To further investigate the potential side effects of SPHF, biological parameters and IHC analysis were examined in xenografted mice. ALT, AST, and ALP levels are established indicators of hepatic function, whereas creatinine and urea levels reflect kidney function. No apparent differences in ALT, AST, ALP, creatinine, or urea serum levels were observed between control and SPHF-treated xenografted mice. Histopathologic examination of the kidney, liver, lung, and heart confirmed that orally administered SPHF did not exert any visible toxic effects. Our data suggest that SPHF is a non-toxic compound with promising antitumor properties, as demonstrated by in vivo studies in tumor-bearing mice. These findings provide preliminary evidence for the development of effective SPHF-based antitumor agents with minimal or no side effects. However, further studies must be conducted to further identify potential adverse effects of SPHF treatment using other toxicity tests.

## 5. Conclusions

This study investigated the antitumor efficacy of fucose-containing sulfated polysaccharides obtained from enzyme-processed HF (SPHF) in bladder cancer in vitro and in vivo. SPHF impeded the proliferation of bladder cancer EJ cells via p21WAF1-mediated G1-phase cell cycle arrest by suppressing CDKs/cyclins expression. Additionally, JNK phosphorylation was induced by SPHF treatment. SPHF-induced the inhibition of EJ cell migration and invasion was attributed to a decrease in transcription factor-associated MMP-9 expression. Moreover, we verified that SPHF suppressed tumor growth in xenografted mice without visible side effects. The inhibitory effect of 20 mg/kg SPHF was found to be equivalent to that of cisplatin (5 mg/kg). Therefore, our findings provide valuable data for the development of novel and effective SPHF-based anticancer agents with minimal side effects.

## Figures and Tables

**Table 1:** Yield, Phenolic content, Sulfated polysaccharide, and monosaccharide content of CHF and SPHF obtained from Hizikia fusiforme.

Sample	CHF	SPHF
Yield (%)	45.12	31.12
Phenolic content (%)	1.88	1.79
Sulfated polysaccharide (%)	51.32	65.23
Proportion of Fucose (%)	27.31	59.63

CHF: Celluclast-assisted extract of Hizikia fusiforme; SPHF: sulfated polysaccharides extracted from celluclast-processed Hizikia fusiforme. The data are presented as the mean ± SD of three independent experiments.

**Table 2:** Free radical scavenging activities of CHF and SPHF obtained from Hizikia fusiforme

Sample	Free Radical Scavenging Activity (IC50 mg/mL)	
	DPPH	TBARs
CHF	0.92 ± 0.03	2.2 ± 0.01
SPHF	0.79 ± 0.04	1.5 ± 0.02

CHF: Celluclast-assisted extract of Hizikia fusiforme; SPHF: sulfated polysaccharides extracted from celluclast-processed Hizikia fusiforme; DPPH:DPPH radical scavenging activity; TBARS: inhibition of lipid peroxidation events. The data are presented as the mean ± SD of three independent experiments.
